# Possible Treatment Approaches of Sulfur Mustard-Induced Lung Disorders, Experimental and Clinical Evidence, an Updated Review

**DOI:** 10.3389/fmed.2022.791914

**Published:** 2022-04-29

**Authors:** Mohammad Reza Khazdair, Mohammad Hossein Boskabady

**Affiliations:** ^1^Cardiovascular Diseases Research Center, Birjand University of Medical Sciences, Birjand, Iran; ^2^Applied Biomedical Research Center, Mashhad University of Medical Sciences, Mashhad, Iran; ^3^Department of Physiology, Faculty of Medicine, Mashhad University of Medical Sciences, Mashhad, Iran

**Keywords:** sulfur mustard (SM), treatment, pharmaceutical drugs, chemical agents, lung injury

## Abstract

Sulfur mustard (SM) is one of the major potent chemical warfare that caused the death of victims in World War I and the Iraq-Iran conflict (1980–1988). The respiratory system is the main target of SM exposure and there are no definitive therapeutic modalities for SM-induced lung injury. The effects of the new pharmaceutical drugs on lung injury induced by SM exposure were summarized in this review. Literature review on PubMed, ScienceDirect, and Google Scholar databases was performed to find papers that reported new treatment approach on SM-exposure-induced injury in the respiratory system until October 2019. The search was restricted to sulfur mustard AND induced injury (*in vitro* studies, animal experiments, and clinical trials) AND respiratory system OR lung, AND treatment in all fields. Two hundred and eighty-three relevant articles were identified that 97 retrieved articles were eligible and were included in the review. Some new pharmaceutical drugs have shown therapeutic potential in controlling various characteristics of lung injury due to SM exposure. Recent studies showed therapeutic effects of mucolytic drugs, non-steroidal drugs, and antibiotics on reducing lung inflammation, oxidative stress responses, and modulating of the immune system as well as improving of respiratory symptoms and pulmonary function tests. Studies on the therapeutic effects of new agents with amelioration or treatment of SM-induced lung injury were reviewed and discussed.

## Introduction

Sulfur mustard (SM) is a toxic vesicant that was first used in 1917 as a chemical weapons (CW) agent and was repeatedly used during Iraq–Iran conflict (1983–1988) (1). For a period (5 years) from August 1983 to July 1988, Iran was attacked several times with CW by the Iraqi army. Iraq used large amounts of CW against Iranian military and civilian people. Sardasht (the Kurdish cities, North West of Iran) was attacked several times in July 1987 and June 1988. The last Iraq chemical attack was in Feb 1988 in the town of Oshnaviyeh (North West of Iran) which injured thousands of civilians. The use of CW against civilian population in eight locations in Oshnaviyeh (Sheikh Othman District) has been confirmed by United Nation’s experts (2).

Sulfur mustard is a cell poison agent which can alter DNA and other nuclear components (3), and cause short and long term injury to heart, lung, nervous, and digestive systems (4, 5) depending on the dose and duration of the exposure (6). The lungs are major targets of SM which led to destruction of bronchial tissues (7), obstruction of airways (8), and oxidative stress (9). Edema and erythema of the pharynx and bronchial tree have occurred minutes to several hours after exposure to SM (10), hemorrhagic pulmonary edema, secondary pneumonia, and respiratory failure 24–48 h after severe exposure (11) and bronchopneumonia 36–48 h after SM exposure (12) in the veterans. Various pathological changes of the lungs (13) in SM exposed individuals can lead to asthma and/or chronic obstructive pulmonary disease (COPD) like symptoms (14).

**FIGURE 1:**
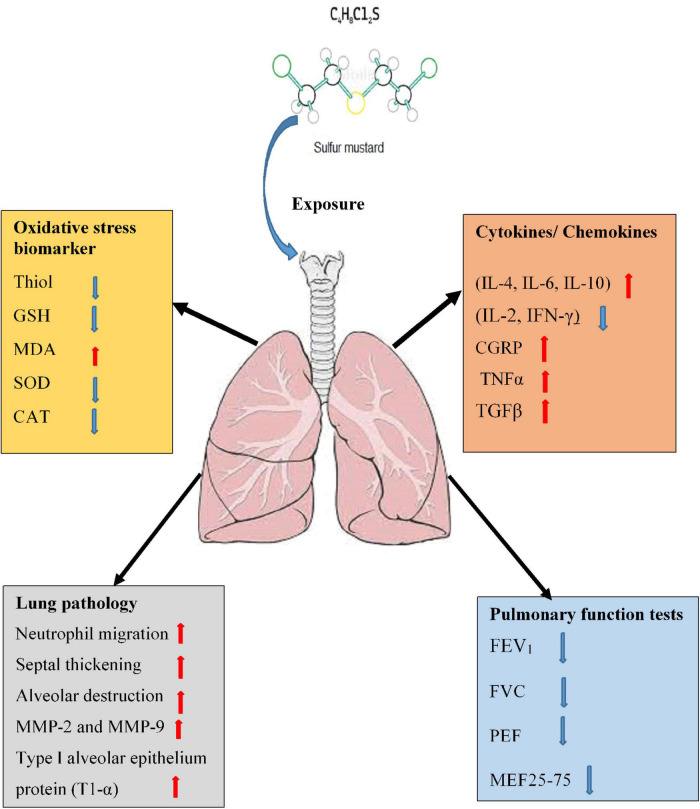
Possible mechanisms of lung injury induced by SM exposure. MDA, malondialdehyde; GSH, glutathione; SOD, superoxide dismutase; CAT, catalase; TGF-β1, transforming growth factor-beta1; IL, interleukin; IFNγ, interferon gamma; TNFα, tumor necrosis factor-α; MMPs, matrix metalloproteinase; FVC, forced volume capacity; FEV1, volume in one second; PEF, peak expiratory flow; MEF, maximal expiratory flow.

Releasing of several inflammatory mediators by phagocytic leukocytes (15, 16), increased bronchoalveolar lavage fluid (BALF) cells (17, 18) which were shown in animals exposed to SM that indicates the role of phagocytic leukocytes and their inflammatory mediators in the pathogenic response to inhaled SM (19). Pulmonary and systemic inflammation such as changes in serum levels of cytokines (20), increased C-reactive protein, (21) and the serum levels of the pro-apoptotic protein, soluble Fas l (22) were observed in individuals long time after exposing to SM. Increased capillary leakage (23) and permeability in the cultured vascular endothelial cell monolayers (24) were also reported due to SM exposure. Hematological changes such as increased WBC count, increased respiratory symptoms, and reduced pulmonary function test (PFT) were reported in veterans long term after SM exposure (25). The possible mechanisms of SM-induced lung disorders were shown in Figure 1.

Presently, there is no approved medication for the treatment of SM exposure-induced lung injury (26), but a combination of vitamin E and corticosteroids was used for protection against acute phases of SM-induced lung injury (27). Moreover, L-nitroarginine methyl ester (L-NAME) and L-thiocitrullline (L-TC) are two protective drugs against the acute toxicity of SM in the *in vitro* and *in vivo* studies (28, 29). The results of these studies showed that different treatments still have no optimal effectiveness and also have known adverse effects in this stage of disease (30). After the acute phase, respiratory problems such as chronic bronchitis (59%), tracheobronchial stenosis (24%), asthma (11%), or bronchiectasis (9%) are the leading causes of long-term disability among patients with exposure to SM (31, 32). Inhaled corticosteroids are extensively used to resolve exacerbation of respiratory symptoms for the treatment of long-term toxic effects of SM (33). In addition, bronchodilators can be applied for treatment of increased airway hyper-reactivity in SM exposed patients (34). The combination of β-agonist and anticholinergic with corticosteroids has been found to be more effective than any of the other bronchodilators used alone in SM exposed patients (35). The possible therapeutic effects of natural products on SM induced complication were also reported (36).

Exposure to SM could induced lung injury including long-term effects like COPD and fibrosis, even decades after exposure. It is essential to identify efficacious treatments for chronic diseases induced by SM. Therefore, this review aimed to present available experimental and clinical publications on the efficacy of the synthesized drugs in the prevention and/or treatment of lung disorders due to SM exposure.

## Possible Treatment Approaches for SM Induced Lung Disorders

### Anti-inflammatory Agents

#### Interferon-Gamma

Interferon-gamma (IFN-γ) and transforming growth factor-beta1 (TGF-β1) showed inhibitory effects on several aspects of the inflammatory process. TGF-β1 induced stimulation of collagen transcription in fibroblasts independent of Stat1–promoter interactions but IFN-γ inhibited these effects (37, 38). IFN-γ and TGFβ may also provide opposing signals to macrophages (39).

##### Experimental Studies

Interferon-gamma modified mesenchymal stem cells (MSCs) induced apoptosis in lung tumor cells through caspase-3 activation. The percentage of activated-caspase-3-positive tumor cells in IFN-γ modified cultured MSCs was significantly higher than in control cultured MSCs. The results of this study provide a new strategy for tumor therapy that utilizes IFN-γ modified MSCs (40).

Anti-inflammatory effect of IFN-γ via down-regulation of TGF-β1 and pro-collagen I and III gene expression in a mouse model of lung fibrosis was demonstrated (41). Interactions of IFN-γ and IL-13, on the mouse model of airway inflammation, showed a negative correlation between increased IFN-γ (number of Th1 cells) with airway hyperreactivity. Also, IFN-γ is able to modulate the effects of IL-13-induced airway hyperreactivity and goblet cell hyperplasia. Intranasal administration of IFN-γ inhibits IL-13-induced goblet cell hyperplasia and airway eosinophilia. Co-administration of IFN-γ and IL-13 showed synergic effects on increased IL-6 level as well as numbers of natural killer (NK) cells and CD11c-positive cells in the airways of mice (42).

##### Clinical Studies

Interferon-gamma has been shown to be effective in the treatment of patients with idiopathic pulmonary fibrosis (IPF) (43). Decreased IFN-γ levels in leukocyte cultures from SM exposed patients were also reported (44). In a clinical study, 36 SM exposed patients with SM bronchiolitis were divided into case and control groups. The case group was treated with a combination of 200 mg IFN-γ and 7.5 mg prednisolone for 6 months. Pulmonary function tests (PFT) including FEV_1_ and forced vital capacity (FVC) were not significantly different at the baseline between the two groups. However, FEV_1_ and FVC were significantly increased in the case group during the subsequent months (45). In another similar study, the effect of interferon IFN-γ on respiratory symptoms, quality-of-life, and oxidative parameters in SM exposed patients was investigated. IFN-γ (100 μg) was administered every other day for 6 months. Severity and frequency of respiratory symptoms, quality-of-life, serum levels of different cytokines, and oxidative stress parameters were assessed at the baseline and at the end of the study. The results showed that IFN-γ therapy not only elevated FEV_1_, but also reduced the severity of cough, dyspnea, and frequency of sputum occurrence. IFN-γ therapy also is associated with improvements in quality-of-life. Serum levels of IL-4, IL-6, IL-10, calcitonin gene-related peptide (CGRP), MMP-9, tumor necrosis factor-alpha (TNFα), TGFβ, and malondialdehyde (MDA) as oxidative stress marker were also decreased while the level of IL-2, IFN-γ, and glutathione (GSH) were increased at the end of study (46).

The above studies suggest the therapeutic potential of IFN-γ in SM-induced lung injury by reduction of inflammatory mediators and oxidative stress as well as respiratory symptoms but increased PFT values in SM exposed patients with lung disorders.

#### Theophylline

Theophylline in high-dose has bronchodilatory effects and in low concentration may have immunomodulatory and anti-inflammatory properties (47). Theophylline suppressed TGF-β-induced type I collagen mRNA expression in lung fibroblasts and also inhibited fetal bovine serum (FBS)-stimulated fibroblast proliferation and TGF-β-induced α-smooth muscle actin protein (48).

##### Experimental Studies

The administration of theophylline (10 or 20 μg/ml) 2 h before human TGF-β1 stimulation suppressed TGF-β-induced type I collagen (COL1) mRNA expression in lung fibroblasts and also inhibited fibroblast proliferation (48). It was reported that theophylline (1–50 mg/kg–1, i.p.) significantly inhibited the inflammation at the early (4 h) and late (48 h) phases of inflammatory reaction induced by carrageenin (1%) in a murine model of pleurisy (49). Theophylline reduced airway inflammation and pathologic changes of lung tissue induced by cigarette smoke in a COPD model of rats (50). Theophylline (3 and 30 mg/kg) reduced the influx of inflammatory cells into the BALF of Guinea pigs exposed to LPS (30 μg/mL). Furthermore, theophylline improved histological changes induced by LPS, including accumulation of inflammatory cells in the lung parenchyma, swelling of the alveolar walls, and goblet cell hyperplasia in the airways (51).

##### Clinical Studies

Treatment of children with moderate to severe cystic fibrosis (CF) by theophylline for 10 days showed protective effects on arterial oxygen desaturation during sleep, increased wakefulness and decreased sleep efficiency (52). Histone deacetylase complex (HDAC) is the main enzyme responsible for regulating inflammatory gene expression activity induced by oxidative stress. Theophylline can restore reduction of this enzyme. Moreover, theophylline may be able to reverse steroid resistance in COPD and other inflammatory lung diseases (53). Treatment of asthmatic smokers with theophylline (400 mg) in combination with inhaled beclometasone (200 mg) per day after four weeks significantly improved peak expiratory flow (PEF) and FVC but a borderline increase in pre-bronchodilator FEV_1_ (54).

In a clinical study, SM-exposed patients were treated with oral slow releasing (SR) theophylline (250 mg), omeprazole (20mg), NAC (600 mg), salmetrol, and fluxitide (2 puffs) twice a day, during 8 weeks of therapy. The results showed that the low dose of theophylline and other mentioned drugs was partially able to decelerate the reductions in PFT values of these patients (55).

The decline of the reductions in PFT values in SM-exposed patients and its effect on CF and improvement of PFT values in asthmatic smokers by theophylline suggest its therapeutic effect on SM-induced lung disorders.

### Protease Inhibitor Agents

#### Doxycycline

Doxycycline (DOX) is an antibiotic used to treat bacterial infections, pneumonia, and other respiratory tract infections that has been reported to exhibit non-specific matrix metalloproteinase (MMPs) inhibitory activity (56). The important role of MMPs in the toxicity of SM in different tissues including, lungs, skin, and eyes were indicated (57).

##### Experimental Studies

It was reported that doxycycline (2 mg/kg, p.o.) reduced lung pathology (neutrophil migration, septal thickening, and alveolar destruction), intra-alveolar inflammatory cells (mainly neutrophils), bacterial number, and MMP-2 and MMP-9 lipopolysaccharide-induced lung injury in C57Bl/6 mice (58). The effects of doxycycline (20 mg/kg or 60 mg/kg, orally), on virulent influenza A/Aichi/2/68 (H3N2) virus-induced lung injury in mice showed a significant decrease in inflammation and protein leakage in the lungs. Treatment with doxycycline also reduced the levels of MMP-2 and MMP-9 activity, T1-α (type I alveolar epithelium protein), and thrombomodulin (endothelial protein) compared to the non-treated group. These results demonstrated that doxycycline treatment is able to reduce lung damage but it did not affect virus titers and body weights (59).

Administration of doxycycline (2 mg/kg) in the drinking water in mice challenged with intratracheal LPS, significantly decreased the number of neutrophils and shed syndecan-1 in BALF. In addition, doxycycline had no significant effect on total BALF protein and the whole lung caspase-3 activity (60). The protective effect of doxycycline on acute lung injury induced by cardiopulmonary bypass (CPB) in 30 healthy mongrel dogs showed that administration of doxycycline (30 mg/kg) in feeding food significantly decreased WBC count, alveolar-arterial oxygen difference, respiratory index, total protein, and myeloperoxidase (MPO) activity in the BALF compared to the control group. Also, doxycycline (60 mg/kg) significantly decreased the concentration of MMP-9 compared to the control group (61).

Administration of doxycycline (2 mg/kg) in drinking water prior to intratracheally administration of bleomycin which induced pulmonary fibrosis, significantly decreased the number of neutrophils, although the total number of cells in the BALF remained unchanged. Dxycycline also attenuated gelatinase activities and reduced production of gelatinase B in BALF (62). Pretreatment of SM exposed guinea pigs with doxycycline resulted in the reduction of gelatinases activity (MMP-2 and MMP-9), decreased lung inflammation (cellularity and protein levels in BAL), and decreased lung pathological changes (epithelial lesions) (63). These results indicated that doxycycline has potent therapeutic effects on LPS and bleomycin-induced pulmonary fibrosis and inflammation in animal models with a similar action to SM-induced lung injury.

##### Clinical Studies

In a clinical study, pharyngeal Chlamydia trachomatis patients were treated with azithromycin (*n* = 78) and doxycycline (*n* = 64). Treatment failure in patients treated with azithromycin and doxycycline was 8.78 (10%) and 1.64 (2%), respectively. Treatment with doxycycline (100 mg) twice a day for 7 days was associated with less treatment failure of oropharyngeal chlamydia compared with azithromycin (1 g), (64). The effect of doxycycline on cystic fibrosis exacerbation in a randomized, double-blind, placebo-controlled study was studied and bio-specimens were collected at the start and the end of the study. Treatment with doxycycline (100 mg) orally twice daily in participants (*n* = 20) was given over an 8-day period during hospitalization, significantly reduced the levels of MMP-9 in sputum, which was also associated with the reduction in active MMP-9 levels (56.5%), and increased TIMP-1 in sputum. Doxycycline also improved forced expiratory volume in the first second (FEV1) and increased the time to next exacerbation compared to the placebo participants (*n* = 19). Furthermore, treatment with doxycycline reduced total hospital days compared with the placebo-treated group (65).

Using nuclear magnetic resonance (NMR)-based metabolomics to obtain serum metabolic profiles of doxycycline-treated (*n* = 60) and standard therapy of COPD patients (*n* = 40), doxycycline (100 mg) significantly increased the values of FEV1/forced vital capacity (FVC) and improved the COPD assessment test (CAT) scores after 3 months’ treatment. In addition, doxycycline significantly down-regulated serum levels of lactate and fatty acid, while, up-regulated the levels of formate, citrate, imidazole, and L-arginine compared to the pre-treatment level. The post doxycycline treatment significantly reduced serum level of the folate compared to COPD patients (66).

The results of experimental and clinical studies indicated that doxycycline could attenuate lung injury and pulmonary edema through anti-inflammatory effects such as degradation of the cell membrane, pulmonary neutrophil infiltration, and PFT test. These results suggest its therapeutic value in COPD and lung injury induced by SM.

#### Tissue Plasminogen Activator

Tissue plasminogen activator (tPA) is a potent fibrinolytic agent that currently used as first-line therapy in clot-associated diseases, such as stroke (67).

##### Experimental Studies

Sulfur mustard analog exposure led to the formation of fibrin-rich in the airways which caused airway obstruction (68). The intra-tracheal administration of tPA (0.15–0.7 mg/kg, 5.5 and 6.5 h) in adult rats exposed to SM analog completely eliminated mortality at 48 h (0%), and improved morbidity (90–100%). Treatment with tPA also normalized plastic bronchitis and hypercarbia. It also improved respiratory distress, pulmonary gas exchange, and oxygen utilization while reduced airway fibrin casts (69).

Intra-tracheal tPA treatment reduced mortality at 48 h (0%) and significantly improved lung injury after lethal SM inhalation (100% death in controls). In addition, tPA improved respiratory distress and normalized hypoxemia, hypercarbia, and lactic acidosis induced by SM. Moreover, tPA was given via airway 6h after SM exposure prevented death from lethal SM inhalation, and normalized oxygenation and ventilation defects (70).

##### Clinical Studies

The effect of intravenous recombinant human tPA (rt-PA) was compared to urokinase in 45 patients with pulmonary embolism (PE). The results showed improvement in lung scan reperfusion in the two treatment groups after 24 h. The reduction in fibrinogen did not differ significantly between the two treated groups. These results also indicated that rt-PA acts more rapidly and is safer than urokinase in the treatment of acute PE (71).

Therefore, tPA reduced mortality and respiratory distress as well as normalized hypoxemia and hypercarbia induced by lethal SM inhalation which suggest the therapeutic effect of tPA on SM-induced lung disorders.

#### Tissue Factor Pathway Inhibitor

Tissue factor pathway inhibitor (TFPI) is a 276 amino acid glycoprotein with three distinct structural domains and an acidic N terminus which is an important physiologic inhibitor of the extrinsic pathway of the coagulation system (72). It was reported that TFPI-2 inhibits tumor invasion and angiogenesis *in vitro* and *in vivo*, and also suggested a potentially important therapeutic role for recombinant TFPI-2 in the treatment of malignant esophageal carcinomas (73).

##### Experimental Studies

Pre and post-exposure treatment of rats with recombinant TFPI (rTFPI) significantly inhibited LPS-induced pulmonary vascular injury and coagulation abnormalities. Treatment with rTFPI also significantly inhibited increases of TNF-α, cytokine-induced neutrophil chemo-attractant, and myeloperoxidase in lung tissue. The administration of rTFPI significantly reduced the expression of TNF-α messenger RNA (mRNA) and inhibited TNF-α production in the lungs after stimulation by LPS. The results of this study suggested the effect of rTFPI on pulmonary vascular injury by inhibiting leukocyte activation in LPS-administered rats (74). Intravenous injection of rTFPI immediately before the introduction of tumor cells also reduced metastasis by 83% in mice (75).

Intra-tracheal administration of TFPI decreased fibrin-containing formation and limited severe hypoxemia in SM analog-induced lung injury in animals. TFPI limited thrombin activation in airways by reduction of prothrombin consumption and decreased thrombin- antithrombin complex (TAT) in BALF which led to a reduced airway obstruction and an improved gas exchange in rats (76). Therefore, the therapeutic effect of TFPI on LPS and SM analog-induced lung injury was documented.

### Antioxidant Agents

#### N-Acetyl Cysteine

N-acetyl cysteine (NAC) is a thiol compound and categorized as a mucolytic drug-containing sulfhydryl groups. It is a reactive oxygen species (ROS) scavenger and also reduces GSH and therefore can regulate the oxidation status in cells. In addition, it interferes with several signaling pathways including regulating apoptosis, cell growth and arrest, angiogenesis, redox-regulated gene expression, and inflammatory response (77).

##### Experimental Studies

The involvement of IL-8 in the pathogenesis of Bleomycin (BLM)-induced lung injury has been suggested and its level was elevated in a bronchial epithelial cell line (BEAS-2B cells). However, BLM-induced expression of IL-8 protein and mRNA in BEAS-2B cells was inhibited by NAC (78). Pre-incubation with NAC (5 × 10^–5^M, 24 h) also significantly reduced peroxynitrite (ONOO–) and O^2–^production in lung macrophages obtained from systemic sclerosis (SSc) patients (79).

Administration of NAC in lead-exposed animals, reduced or reversed lead-induced oxidative stress. Lead-exposed rats resulted in signs of anemia such as anisocytosis, poikilocytosis, and alterations in hemoglobin and hematocrit. It also causes an alteration in lipid peroxidation such as, increased MDA content and a decrease in GSH which are reversed by NAC (80). It has been reported that pretreated animals with NAC or liposomally-entrapped *N*-acetylcysteine (L- NAC) (25 mg/kg, iv), and challenged with lipopolysaccharide (LPS) (*Escherichia coli*, LPS 0111:B4) prevents increased lung weights, decreased lung angiotensin-converting enzyme (ACE) (the injury marker for pulmonary endothelial cells), and significantly reduced the LPS-induced increase in plasma TNF-α levels. In addition, NAC significantly decreased myeloperoxidase (MPO) and chloramine concentrations in the lungs and the extent of lipid peroxidation in liver tissues (81).

The enhancement of endothelial survival via GSH against SM injury of the endothelium was also reported. In this study, exposure to 500 μM SM resulted in increased activation of the nuclear transcription factor (NF_kB_) binding to its consensus sequence 5 h after exposure. Pretreatment with NAC suppressed SM-induced NF_kB_ activation which is an important transcription factor for a number of cytokine genes (e.g., TNF) and is activated following stress in endothelial cells (82). GSH-induced enhancement of cell viability at 2.5 and 5 μM was shown after SM exposure. Pretreatment with hioninesulfoximine (BSO) an inhibitor of GSH synthesis, alone did not show toxicity effect but potentiated the toxicity of SM (82). Similarly, SM exposure led to a dose and time-dependent decrease in GSH content in human skin fibroblast cell line (HF2FF). NAC increased intracellular GSH level and protected the cells against SM-induced reactive oxygen species formation and lactate dehydrogenase leakage. In contrast, BSO pretreatment reduced cellular GSH and enhanced the cytotoxic effects of SM on HF2FF cells (83).

Treatment with intra-tracheal administration of NAC on pulmonary edema formation induced by phosgene exposure in rabbit lungs lowered pulmonary artery pressure, lung weight gain, the concentration of peptide leukotrienes LTC_4_, D_4_, and E_4_, and lipid peroxidation which were increased following exposure to phosgene (84).

Animal exposure to SM leads to inflammatory cell accumulation in the airways and lung as well as structural and functional alterations in the respiratory tract (18, 85). The role of oxygen species and free radicals in the pathophysiology of inflammation-induced pulmonary lesions was described previously (86). However, treatment with NAC reduced the number of neutrophils in mice that were exposed to SM and developed lung injuries (87). Following animal exposure to SM vapor (100 μg/kg) for 10 min, arterial blood oxygen saturation levels (SaO_2_), arterial blood pH and bicarbonate (HCO${}_{3}^{-}$) were significantly decreased. Also, arterial blood carbon dioxide (PaCO_2_) and shunt fraction were significantly increased. After treatment with inhaled doses of NAC (1 ml of 200 mg) arterial blood oxygen saturation, HCO${}_{3}^{-}$ level, and shunt fraction were significantly improved compared to those of the SM exposure. In addition, infiltration of neutrophils and concentrations of protein in BALF were significantly decreased compared to the SM group (88).

##### Clinical Studies

In clinical studies, treatment with NAC in patients with COPD, asthma, and acute bronchitis improved respiratory symptom, lung function, and quality of life. Treatment with NAC also scavenged reactive oxygen species and inhibited mediator release (89, 90). It was reported that the administration of NAC, 600 mg daily for 12 months in patients with COPD, reduced oxidative stress (91, 92) and also the oxidative burst of poly morphonuclear (PMN) cells and showed protective effects on peripheral granulocytes in COPD patients (93, 94).

Intravenous administration of NAC (190 mg/kg/day) in adult patients (*n* = 42) with respiratory distress syndrome (Pao2/Fio2 ≤ 200 mm Hg) significantly decreased the lung injury score between days 1 and 3 compared to the placebo group (95).

Treatment with NAC (40 mg/kg/day) intravenously for 3 days in 61 adult patients with mild-to-moderate acute lung injury (NAC, *n* = 32 and the placebo groups, *n* = 29 patients) improved ventilation compared to the placebo group. The oxygenation index (PaO_2_/FIo_2_) significantly was improved from day 0 to day 3 only in the NAC treated group and the lung injury score was improved in the NAC treated group during the first 10 days of treatment but, no change was observed in the placebo group (96).

Treatment of SM-induced bronchiolitis obliterans patients (*n* = 144) with NAC (1,200 mg daily), improved dyspnea, wake-up dyspnea, and cough after 4 months of treatment compared to the control group. Additionally, NAC reduced sputum from (76.9%) of cases before the study to (9.6%) of cases at the end of trial. The values of FEV1, FVC, and FEV1/FVC were also significantly improved in NAC treated patients compared to the placebo group (97).

In another clinical study, treatment with NAC (1,800 mg daily) in 144 bronchiolitis obliterans patients 18 years after SM exposure improved clinical signs and symptoms of patients including; dyspnea, cough, sputum, wake-up dyspnea, and also increased PFT values (98). It has been reported that administration of NAC (oral, IV, IP or IT) is effective in the management of SM induced acute lung injury due to inhibition of oxidative stress, inflammatory responses and apoptosis in a time-dependent manner. Furthermore, oral NAC alone at high doses for 4 months or in combination with clarithromycin (500 mg/day) at a dose 600 mg/day for 6 months improved clinical and paraclinical pulmonary parameters of patients with bronchiolitis obliterans induced by SM exposure (99). The efficacy of NAC in reducing SM toxicity in different studies (*in vitro* and *in vivo*) model of lung injury as well as the safe and efficacy of NAC in treating patients suffering the long-term and chronic pulmonary effects of SM exposure in veterans was reported (100).

Both experimental and clinical studies suggest the possible therapeutic effect of NAC on SM-induced lung disorders and other respiratory diseases via possible anti-inflammatory and anti-oxidant mechanisms.

#### Vitamin E

##### Experimental Studies

The effects of vitamin C, vitamin E, and rINN, alone or in combination in placental villi culture after exposure to nicotine-induced endothelial dysfunction reduced placental cell proliferation until cell death (101). Vitamin E reduced phospholipidosis in cultured human skin fibroblasts chronically exposed to amiodarone and desethylamiodarone (DEA) and inhibited cumulative uptake of the drugs in a dose-dependent manner (102). Vitamin E pretreatment on human fibroblast cells significantly protected against ciprofloxacin (CPFX)-induced cytotoxicity. It also significantly increased the total GSH content and reduced the level of lipid peroxidation (103).

The effects of liposome-encapsuled vitamin E on a mouse model for airway inflammation induced by inhalation exposure to the alkylating nitrogen mustard melphalan showed that vitamin E (50 mg/kg) reduced inflammatory cell influx, and inhibited collagen formation in lung tissue (27). The administration of vitamin E (600 mg/kg), dexamethasone (5 mg/kg) or their combination in guinea pigs exposed to SM caused improvement in the pathological changes in the livers and kidneys (104).

The effect of vitamin E on tracheal responsiveness (TR) and lung inflammation in SM exposed animals also showed that TR to methacholine, total and differential WBC count in lung lavage, and serum levels of IL-4 were significantly decreased in treated animals with vitamin E compared to the untreated SM-exposed group (105). In addition, vitamin E treatment reduced pathological changes in guinea pigs exposed to SM. The antioxidant effect of tocopherol acetate (vitamin E) was also reported (106, 107) which supports the above results.

Tocopherol acetate (200 mg/kg, i.p.) increased GSH levels and decreased MDA level in systemic toxicity due to percutaneous administration of SM in animals. Furthermore, total antioxidant status was increased but red blood corpuscles and hemoglobin content were significantly decreased in the tocopherol acetate treated group (108).

Liposomes containing tocopherol (α, γ, δ) showed protective effects on the accumulation of RBC in the bronchi, alveolar space, arterioles and veins, and fibrin and collagen deposition in the alveolar space on 2-chloroethyl ethyl sulfide (CEES) (a mono-functional analog of sulfur mustard)-induced lung injury in a guinea pig. Moreover, tocopherol decreased lipid peroxidation level and hydroxyproline in the lungs (109). The treatment with liposome of tocopherol (α, γ, δ) significantly blocked the CEES-induced increase in the protein levels of the cell cycle protein (cyclin D1), and a cell differentiation marker (PCNA). Additionally, tocopherol protected the lungs against CEES-induced inflammation and alveolar infiltration of neutrophils and eosinophils (110).

The effect of vitamin E on CEES and alkylating nitrogen mustard-induced lung inflammation, as well as its effect on lung oxidative stress, lung inflammation, lung pathology, and tracheal responsiveness due to similar mechanisms in SM-exposed subjects indicated its therapeutic value in SM and other chemical-induced lung injuries.

##### Clinical Studies

Dietary intakes of vitamin E in 178 men and women aged (70–96 year) indicated that intake of vitamin E may influence lung function by increasing FVC, FEV1 and showed protective effects on wheeze in elderly in three European countries including Finland (*n* = 1,248), Italy (*n* = 1,386), and the Netherlands (111, 112). Treatment of 33 mild atopic asthmatics subjects for 16 weeks, with high doses of -α-tocopheryl acetate significantly decreased F2-isoprostanes, and reduced allergen-provoked concentrations of IL-3 and IL-4, and increased levels of IL-12 in BALF. In addition, natural source of vitamin E improved airway responsiveness to methacholine (113).

In a placebo-controlled randomized clinical trial the effects of natural vitamin E (500 mg) or matched placebo in 72 participants aged (18–60 years) for 6 weeks, had no significant impact on FEV1, asthma symptom scores, or serum immunoglobulin levels (114). Investigation of the influence of maternal antioxidant intake in pregnancy on the development of asthma and eczema in children showed that maternal vitamin E intake during pregnancy is negatively associated with wheeze in the absence of “cold” and childhood eczema in the children’s with 24 months of age (115).

**FIGURE 2:**
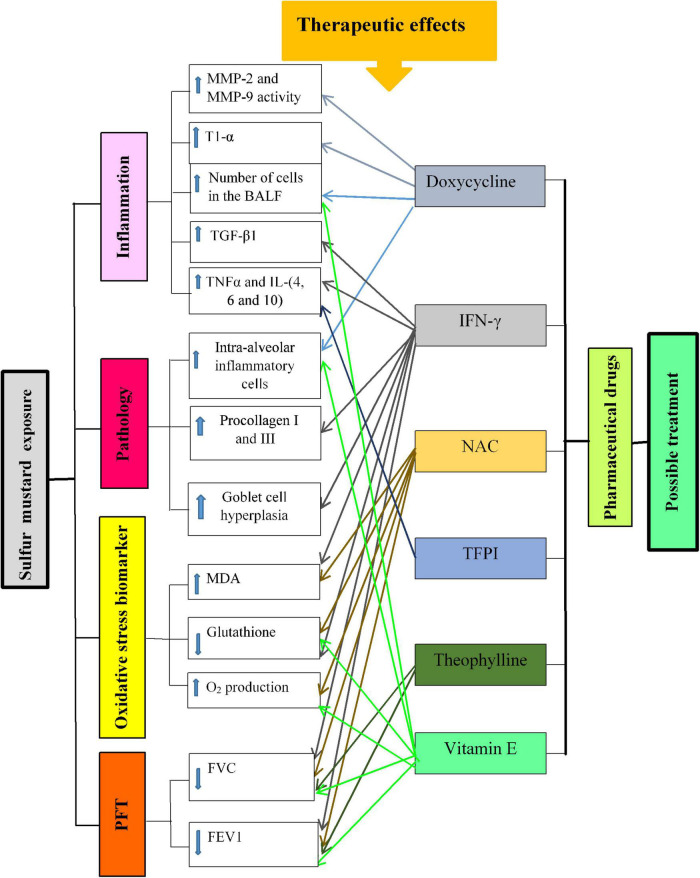
Possible mechanisms of lung injury induced by SM exposure and the effect of pharmaceutical drugs in these changes. T1-α, type I alveolar epithelium protein; MMPs, matrix metalloproteinase; BALF, bronchoalveolar lavage fluid; TGF-β1, transforming growth factor-beta1; IL, interleukin; IFNγ, interferon gamma; TNFα, tumor necrosis factor-α; NO, nitric oxide; MDA, malondialdehyde; FVC, forced volume capacity; FEV1, volume in one second.

The therapeutic effects of pharmaceutical drugs on SM-induced lung disorders in experimental and clinical studies were summarized in Tables 1, 2, respectively. The possible mechanisms of the therapeutic effects of pharmaceutical drugs on SM-induced lung disorders were also summarized in Figure 2.

**TABLE 1:** Therapeutic effects of pharmaceutical drugs on SM-induced lung injuries, experimental evidence.

Drugs		Agent induced lung injury	Model of study	Effects	Ref.
Anti-inflammatory agents	Gamma-interferon	Lung tumor	MSCs cells	Enhanced activated-caspase-3-positive tumor cells in IFN-γ modified cultured MSCs	(40)
		Bleomycin	Mouse	Down-regulated of TGF-β1 and procollagen I and III gene expression	(41)
		Mixed T cell	Mouse	Inhibited IL-13-induced goblet cell hyperplasia and airway eosinophilia	(42)
	Theophylline	Fetal bovine serum (FBS)	Fibroblasts	Suppressed TGF-β-induced type I collagen mRNA expression, inhibited fibroblast proliferation	(48)
		Carrageenin	Mice	Inhibited inflammation at the early (4 h) and late (48 h) phases of inflammatory reaction	(49)
		COPD	Rats	Reduced airway inflammation and pathologic changes of lung tissue	(50)
		LPS	Guinea pigs	Reduced inflammatory cells into the BALF, improved lung histological changes induced by LPS	(51)
Protease inhibitors	Doxycycline	LPS	Mice	Reduced lung pathological changes, intra-alveolar inflammatory cells, MMP-2, and MMP-9	(58)
		Virulent A/Aichi/2/68 (H3N2)	Mice	Decreased inflammation and protein leakage in the lungs, reduced MMP-2 and MMP-9 activity	(59)
		Bleomycin	Mice	Decreased neutrophils, gelatinase activities, and production of gelatinase B in BALF	(62)
		LPS	Mice	Decreased neutrophil and shed syndecan-1 in BALF	(60)
		Cardiopulmonary bypass	Dog	Decreased WBC count, alveolar-arterial oxygen difference, respiratory index, total protein, and MPO activity in the BALF	(61)
		SM	Guinea pigs	Reduced activity of MMP-2 and MMP-9, and lung inflammation	(63)
	tPA	SM	Rat	Eliminated mortality at 48 h (0%), normalized plastic bronchitis, and hypercarbia, improved respiratory distress, pulmonary gas exchange, and oxygen utilization, reduced airway fibrin casts	(69)
		SM	Rat	Reduced mortality (0% at 48 h), improved lung injury, improved respiratory distress and normalized hypoxemia	(70)
	TFPI	LPS	Rats	Inhibited pulmonary vascular injury coagulation abnormalities and rTFPI, increased TNF-α	(74)
		-	Mice	Reduced metastasis by 83%	(75)
		SM	Rat	Limited thrombin activation in airways, decreased thrombin anti-thrombin complex in BALF	(76)
Antioxidants agents	N-acetylcysteine	Bleomycin	BEAS-2B cells	Inhibited expression of IL-8 protein and mRNA	(78)
		-	Lung macrophages	Reduced peroxynitrite (ONOO–) and O2-production	(79)
		Lead	Rats	Reversed lead-induced alterations in MDA and GSH content	(80)
		LPS	Rodents	Decreased MPO and chloramine concentrations in the lungs and lipid peroxidation in liver tissues	(81)
		SM	Endothelial cells	Enhances endothelial cells survival, suppressed SM induced activation of NF_kB_ at 5 h	(82)
		SM	(HF2FF) cells	Increased intracellular GSH level and protected the cells against reactive oxygen species formation and lactate dehydrogenase leakage	(83)
		Phosgene	Rabbit	Decreased pulmonary arterial pressure, lung weight gain, peptide leukotrienes LTC_4_, D_4_, and E_4_, and lipid peroxidation levels	(84)
		SM	Mice	Reduced neutrophils and developed lung injuries	(87)
		SM	Pigs	Improved arterial blood oxygen saturation, HCO${}_{3}^{-}$ levels, and shunt fraction	(88)
	Vitamin E	CPFX	Fibroblasts	Increased total GSH content and reduced lipid peroxidation level	(103)
		Nitrogen mustard	Mic	Reduced inflammatory cell influx, inhibited collagen formation in lung tissue	(27)
		SM	Guinea pigs	Improved pathological changes in the livers and kidneys	(104)
		SM	Guinea pigs	Decreased TR to methacholine, total and differential WBC and IL-4	(105)
		SM	Guinea pigs	Decreased lung pathological changes	(107)
		SM	Mic	Increased GSH levels, decreased MDA level and total antioxidant status	(108)
		CEES	Guinea pigs	Decreased level of lipid peroxidation and hydroxyproline in the lung	(109)
		CEES	Guinea pigs	Protected lung against CEES-induced inflammation, decreased neutrophils and eosinophils in the alveolus	(110)

*SM, sulfur mustard; COPD, chronic obstructive pulmonary disease; BALF, broncho-alveolar lavage fluid; WBC, white blood cells; IFNγ, interferon-gamma; TGF-β1, transforming growth factor beta1; IL, interleukin; NK cells, natural killer cells; TNFα, tumor necrosis factor-α; NAC, N-acetylcysteine; LPS, lipopolysaccharides; tPA, tissue plasminogen activator; MDA, malondialdehyde; GSH, glutathione; MPO, myeloperoxidase; CEES, 2-chloroethyl ethyl sulfide; BM, basement membrane.*

**TABLE 2:** Therapeutic effects of pharmaceutical drugs on SM-induced lung injuries, clinical studies.

Drugs		Studied agent	Model of study	Effects	Ref.
Anti-inflammatory agents	Gamma-interferon	SM	Human	Increased FEV1 and FVC values	(45)
		SM	Human	Improved FEV1, cough, dyspnea, morning dyspnea, sputum, and IL-4, IL-6, IL-10, CGRP, MMP-9, TNFα, TGFβ, and MDA serum levels	(46)
	Theophylline	Cystic fibrosis	children	Increased, wakefulness, decreased sleep deficiency and arterial O2 desaturation	(52)
		COPD	Human	Reverse steroid resistance	(53)
		-	Human	Improved PEF, FVC, and FEV1	(54)
		SM	Human	Decelerate the reductions rate of PFT values	(55)
Protease inhibitors	Doxycycline	Cystic fibrosis	Human	Improved sputum MMP-9 and TIMP-1, improved FEV1, and exacerbation	(65)
		COPD	Human	Improved FEV1/FVC value and the COPD assessment test (CAT) scores	(66)
		SM	Human	Decreased treatment failure	(64)
	tPA	Pulmonary embolism	Human	Improved lung scan reperfusion	(71)
Antioxidants agents	N-Acetylcysteine	RDS	Human	Decreased the lung injury score between days 1 and 3 compared to the placebo group	(95)
		Acute lung injury	Human	Improved lung injury score and regression compared to the placebo group	(96)
		Asthma and COPD	Human	Reduced respiratory symptoms and improves PFTs values and quality of life	(89, 90)
		COPD	Human	Reduced oxidative stress in patients with COPD	(91, 92)
		COPD	Human	Reduce the oxidative burst of PMN cells	(93, 94)
		SM	Human	Improved clinical signs and symptoms and PFT values	(98)
		SM	Human	Improved respiratory symptoms, reduced sputum formation, improved FEV1, FVC, and FEV1/FVC values	(97)
	Vitamin E	–	Human	Increased FVC, FEV1, and improved wheeze in elderly in three European countries	(111, 112)
		Asthma	Human	Decreased F2-isoprostanes, allergen-provoked IL-3 and IL-4 levels and AR, augmented IL-12 in the BALF	(113)
		–	Human	Negative association of maternal vitamin E intake during pregnancy with wheeze and childhood eczema	(115)

*SM, sulfur mustard; COPD, chronic obstructive pulmonary disease; RDS, respiratory distress syndrome; PFT, pulmonary function tests; FVC, forced vital capacity; FEV1, forced expiratory volume in the first second; PEF, peak expiratory flow; BALF, broncho-alveolar lavage fluid; WBC, white blood cells; IFNγ, interferon-gamma; TGF-β1, transforming growth factor beta1; IL, interleukin; TNFα, tumor necrosis factor-α; NAC, N-acetylcysteine; LPS, lipopolysaccharides; tPA, tissue plasminogen activator; MDA, malondialdehyde; GSH, glutathione; CGRP, calcitonin gene-related peptide; AR, airway responsiveness.*

## Conclusion

Sulfur mustard, CEES, and nitrogen mustard exposure due to similar actions induces various acute and chronic diseases such as lung disorders. Unfortunately, there is still no definitive medication for SM-induced diseases in clinics. However, several studies suggested the therapeutic effects of individual or a combination of pharmaceutical drug products on SM and its analogs-induced lung disorders in animals as well as in some clinical studies. In this review, published papers regarding the new approach for the treatment of SM-induced lung disorders by pharmaceutical drugs were discussed.

The results of the reviewed papers showed that pharmaceutical drugs improved inflammatory cells, inflammatory mediators, oxidative stress biomarkers, and lung pathological changes in lung injury induced by various agents including SM exposure. The pharmaceutical agents also showed a reduction of clinical symptoms, improvement of PFT values, and health-related quality of life as well as systemic and lung inflammation and oxidative stress in patients suffering from various chronic lung diseases including SM-induced pulmonary complications. Therefore the present review present various potential treatment strategy for preventing and treatment of lung injury patients who are exposed to SM in mean of inflammation and oxidative stress. However, more studies especially clinical trials are needed to examine the exact mechanisms of action of pharmaceutical drugs and their long-term outcome as well as exploring new drugs for the treatment of the serious condition of lung disorders induced by SM exposure.

## Author Contributions

MRK searched the literature and wrote the first draft of the manuscript. MHB designed the study and critically edited and revised the manuscript. Both authors contributed to the article and approved the submitted version.

## Conflict of Interest

The authors declare that the research was conducted in the absence of any commercial or financial relationships that could be construed as a potential conflict of interest.

## Publisher’s Note

All claims expressed in this article are solely those of the authors and do not necessarily represent those of their affiliated organizations, or those of the publisher, the editors and the reviewers. Any product that may be evaluated in this article, or claim that may be made by its manufacturer, is not guaranteed or endorsed by the publisher.

## Abbreviations

Ach: acetylcholine
BALF: broncho-alveolar lavage fluid
CGRP: calcitonin gene-related peptide
COPD: chronic obstructive pulmonary disease
DOX: doxycycline
FDA: Food and Drug Administration
FEV_1_: forced expiratory volume in the first second
FVC: forced vital capacity
GSH: glutathione
HDAC: histone deacetylase complex
hPBMCs: human peripheral blood mononuclear cells
HRQoL: health-related quality of life
hs-CRP: high-sensitivity C-reactive protein
IFNγ: interferon-gamma
IL: interleukin
iNOS: induced nitric oxide synthase
IPF: idiopathic pulmonary fibrosis
LPS: lipopolysaccharides
MCHC: mean corpuscular hemoglobin concentration
MCP-1: monocyte chemotactic protein-1
MCV: mean corpuscular volume
MDA: malondialdehyde
MMEF: maximum mid-expiratory flow
MMPs: matrix metalloproteinase
NAC: N-Acetylcysteine
NFkB: nuclear transcription factor
NK cells: natural killer cells
OVA: ovalbumin
PaCO_2_: arterial blood carbon dioxide
PE: pulmonary embolism
PEF: peak expiratory flow
PFT: pulmonary function tests
ROS: reactive oxygen species
SaO_2_: arterial blood oxygenation saturation
SIL-BS: silibinin-bis-succinat
SM: sulfur mustard
TAT: thrombin anti-thrombin
TFPI: tissue factor pathway inhibitor
TGF-β1: transforming growth factor beta1
TNFα: tumor necrosis factor-α
tPA: tissue plasminogen activator
TR: tracheal responsiveness
TSM: tracheal smooth muscle
WBC: white blood cells.
